# PD-L1 and intratumoral immune response in breast cancer

**DOI:** 10.18632/oncotarget.18305

**Published:** 2017-05-30

**Authors:** Zhi-Qiang Wang, Katy Milne, Heather Derocher, John R. Webb, Brad H. Nelson, Peter H. Watson

**Affiliations:** ^1^ Trev & Joyce Deeley Research Centre, British Columbia Cancer Agency, Victoria, British Columbia, Canada; ^2^ Department of Biochemistry and Microbiology, University of Victoria, Victoria, British Columbia, Canada; ^3^ Medical Genetics, University of British Columbia, Vancouver, British Columbia, Canada; ^4^ Department of Pathology and Laboratory Medicine, University of British Columbia, Vancouver, British Columbia, Canada

**Keywords:** PD-L1, CD4, CD8, CD68, breast cancer

## Abstract

**Purpose:**

PD-L1 is thought to play an important role in the antitumor immune response. In this study, we investigated the expression of PD-L1 within breast tumor subsets to better define its prognostic significance.

**Methods:**

Immunohistochemistry was performed to determine PD-L1 tumor cell expression and to enumerate CD8, CD4 and CD68 tumor-infiltrating leucocytes (TIL) in a cohort of 443 breast cancers categorized by molecular subtype.

**Results:**

Across the entire cohort, PD-L1 tumor cell expression was observed in 73/443 (16.5%) cases and associated with known indicators of poor prognosis, including low patient age, high tumor grade, ER/PR negative status, but not with outcome. However, in the Triple Negative breast cancer subset PD-L1 was associated with better recurrence free survival (RFS) especially within the Basal-like subset (Hazard ratio = 0.39, 95% CI = 0.22 - 0.86, *p* = 0.018). Combined PD-L1/epithelial CD8 positive status was also strongly associated with better RFS and OS (Hazard ratio = 0.12, 95% CI = 0.10 - 0.71, *p* = 0.010 and Hazard ratio = 0.11, 95% CI = 0.11 - 0.68, *p* = 0.006 respectively) in the Basal-like subgroup.

**Conclusions:**

PD-L1 expression is associated with better patient survival in Basal-like breast cancer.

## INTRODUCTION

PD-L1 (B7-H1, CD274) is the ligand for Programmed death-1 (PD-1, CD279) and is expressed on the surface of cancer cells in addition to its expression on infiltrating immune cells. PD1 is an immune-suppressive receptor that is expressed on activated T cells, B cells, monocytes and dendritic cells [1, 2]. PD-L1 activates PD1 leading to inhibitory signals that regulate T-cell activation and tolerance [1] and impede the antitumor immune response [3, 4].

Recent research has shown that blockade of the interaction between PD-L1 and PD-1 can enhance T cell function and facilitate antitumor activity, and various monoclonal antibodies against PD-1 and PD-L1 are in clinical trials for a variety of solid tumor types, including breast cancer, with encouraging activity in many cancers [5]. PD-L1 expression has been studied as a potential biomarker of response in different types of cancer [6–14]. However, the prognostic value of high PD-L1 expression in malignancies remains unclear: most studies reveal a correlation with worse outcome [7–11], whereas a correlation with favorable outcome has been observed in ovary, melanoma, glioma and non-small cell lung carcinoma [12–15]. The results of breast cancer studies are no different. Some studies revealed a negative correlation between PD-L1 expression and outcome [16, 17], while others showed no association with outcome [18, 19] or a positive association with outcome [20–23].

These conflicting results warrant further exploration. When reviewing the literature, we observed that few previous studies had been conducted on the Basal-like subset of breast cancer (see Supplementary Table 1). This is important because evidence shows that the association between tumor infiltrating leucocytes (TILs) and survival is strongest in this Triple Negative tumor subset, suggesting that this is the most immunologically reactive form of breast cancer. Therefore, there is a clear need for studies with sufficient cohort size and appropriate categorization to enable major subgroup analysis to evaluate the significance of PD-L1 in subgroups of breast tumors. The primary aim of this study was to investigate the association of PD-L1 expression on tumor cells with clinical outcome in a large population-based cohort with a long-term follow-up and within five recognized molecular subsets of breast cancer. We also analyzed the relation between PD-L1 expression and three key indicators of the intra-tumoral immune response, CD8, CD4, and CD68 tumor infiltrating leucocytes (TIL).

## RESULTS

### Cohort characteristics

We studied a cohort of 443 patients with primary breast cancer diagnosed in the period 1988-1995. The mean length of follow-up data was 87 months (range 2 to 251 months). There were 184 breast cancer-specific deaths (mean time from diagnosis = 26 months) and 259 survivors (mean time to last follow-up date = 90 months). Primary therapy included surgical resection in all cases, followed by adjuvant hormone, radiation, and chemotherapy in 331 (75%), 160 (36%), 93 (21%) cases, respectively; 30 cases (7%) did not receive any form of systemic therapy. The clinical-pathological characteristics of the entire cohort and the Basal-like subgroup are provided (Table 1).

**Table 1:** Demographic and clinical-pathological characteristics of patients in the study cohort

Parameter	Status	Total cohort		Basal subset	
		Cases	%	Cases	%
Age at diagnosis	≤35 years	13	3	7	10
	>35years	430	97	62	90
Tumor size**^a^**	T1a/b	2	<1	1	<1
	T1c	69	16	7	10
	T2	277	63	40	58
	T3	66	15	15	22
	Unknown	29	7	6	9
Nodal status	Positive	190	43	33	48
	Negative	228	51	26	38
	Unknown	25	6	10	14
Tumor grade	1	71	16	4	5
	2	267	60	19	28
	3	103	23	46	67
	Unknown	2	<1	0	0
ER**^b^**	Positive	203	46	0	0
	Negative	240	54	69	100
	Unknown	0	0	0	0
PR**^b^**	Positive	228	51	0	0
	Negative	215	49	69	100
	Unknown	0	0	0	0
Molecular subtypes	Luminal A	193	44		
	Luminal B	48	11		
	Her2	67	15		
	TNNB**^c^**	35	8		
	Basal-like	69	15		
	Unclassified	32	7		

^a^Tumor size: 0.1cm<T1a/b<1cm; 1cm ≤ T1c<2cm; 2cm≤T2<5cm; 5cm≤T3.

^b^ER negative defined as <10 fmol/mg protein and PR negative as ≤15 fmol/mg protein (ligand binding assay).

^c^TNNB: Triple negative-non-basal.

### Expression of PD-L1 and association with clinical-pathological features

**Figure 1:**
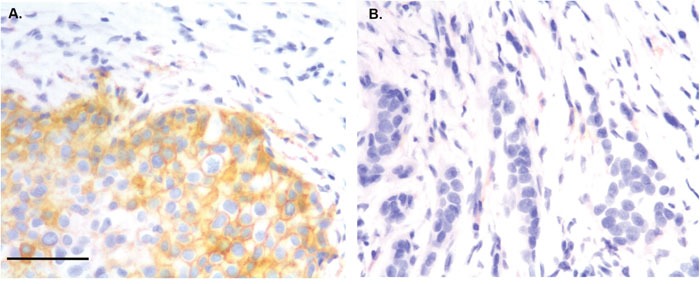
Immunohistochemical staining showing PD-L1 positive staining in tumor cells **(A)** and TIL **(B)**. Magnification ×400. Bar 200 um.

We examined the association between tumor cell PD-L1 expression and clinical-pathological features using an FDA approved and validated antibody and a defined cutoff value of the 75^th^ percentile of the range of scores to delineate low from high expression levels. Expression of PD-L1 by tumor cells was observed in 73 (16.5%) of the 443 evaluable primary breast cancers (Figure 1). The typical PD-L1 tumor cell staining pattern observed was membranous. PD-L1 staining was also frequently observed in stromal cells with features suggesting macrophage like cells (Figure 1) as previously described in ovarian cancer, but this staining not scored [15]. PD-L1 expression was significantly associated with patient age, tumor grade, and ER/PR status (Table 2). PD-L1 positive status was also significantly different between the molecular intrinsic subtypes: high levels of PD-L1 were seen in only a small proportion of Luminal A, Luminal B, and Her2 subtype tumors (12% and 9% respectively) and an intermediate proportion of Luminal B subtype tumors (21%) compared to higher proportions of Triple Negative Non-Basal (TNNB) and Basal-like subtype tumors (31% and 33% respectively; Table 2).

**Table 2:** Association between PD-L1 expression and clinico-pathological characteristics

Parameter		PDL-1 expression		
		Low (%)	High (%)	*p*-value
Age at diagnosis (yrs)	≤35 years	8 (62%)	5 (38%)	**0.046**
	>35 years	362 (84%)	68 (16%)	
Tumor size (cm)	T1a/b	1 (50%)	1 (50%)	0.629
	T1c	57 (83%)	12 (17%)	
	T2	231 (83%)	46 (17%)	
	T3	56 (85%)	10 (15%)	
Nodal status	Positive	193 (85%)	35 (15%)	0.980
	Negative	161 (85%)	29 (15%)	
Tumor grade	1	62 (87%)	9 (13%)	**0.001**
	2	233 (87%)	34 (13%)	
	3	73 (71%)	30 (29%)	
ER status	Positive	212 (88%)	28 (12%)	**0.005**
	Negative	158 (78%)	45 (22%)	
PR status	Positive	192 (89%)	23 (11%)	**0.002**
	Negative	178 (78%)	50 (22%)	
Molecular subtypes	Luminal A	170 (88%)	23 (12%)	**0.013**
	Luminal B	38 (79%)	10 (21%)	
	Her2	61 (91%)	6 (9%)	
	TNNB**^a^**	24 (69%)	11 (31%)	
	Basal-like	46 (67%)	23 (33%)	

^a^TNNB: Triple negative non-basal.

### Correlation between tumor PD-L1 and pattern of the intra-tumor immune response

The relation between PD-L1 and the levels and localization of CD8, CD4, and CD68 TIL was assessed (Figure 3). As reported in our prior studies [24, 25], the intraepithelial densities of all three TIL subsets were higher in triple negative subsets (TNNB and Basal-like) compared to the rest of the cohort. PD-L1 positive status tended to be associated with higher TIL density in all subsets. This pattern was most prominent for intra-epithelial CD8 and CD68 in the triple negative subsets compared to the non-TNBC subsets (Figure 3A and 3B). However, despite the differing prognostic significance of PD-L1 within the two TNBC subsets (Basal-like and TNNB tumors), the overall pattern of TIL was no different in PD-L1 positive and negative cases (Figure 3C and 3D).

**Figure 2:**
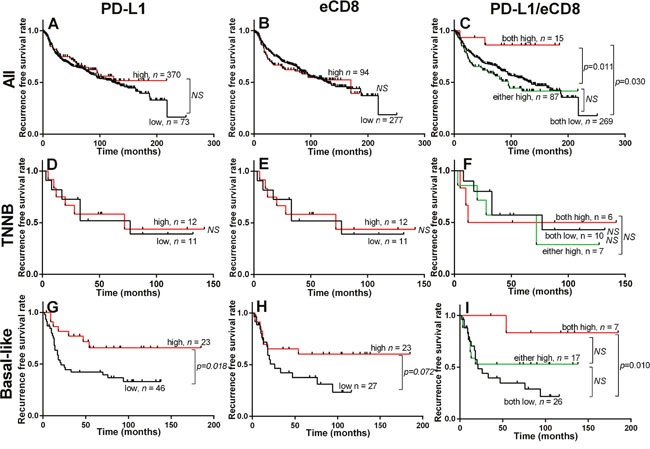
Prognostic impact of PD-L1 and CD8 in breast cancer Kaplan–Meier plots showing recurrence free survival (RFS) in entire cohort, TNNB and Basal-like subgroups stratified according to the expression status of intra-epithelial PD-L1 **(A, D, G)** and eCD8 **(B, E, H)** and PD-L1/eCD8 in combination **(C, F, I)**. The log-rank test was used to compare curves, and *p*-values less than 0.05 were considered significant. NS = no significance.

**Figure 3:**
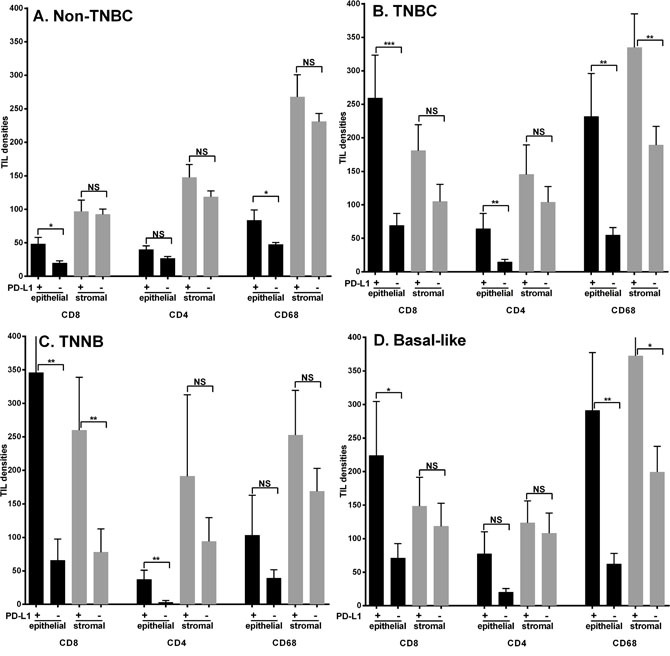
Associations between tumor cell PD-L1 expression and TIL cell densities within Non-TNBC **(A)**, TNBC **(B)**, TNNB **(C)** and Basal-like subtypes **(D)**. Bars represent means +/- standard error. Black and grey bars represent intra-epithelial and intra-stromal densities of CD8, CD4, and CD68 positive TIL respectively. Non-TNBC = Luminal A + Luminal B + Her2; TNBC cases = TNNB (Triple Negative Non Basal) + Basal-like subtypes; NS = no significance, *p ≤ 0.05, ** *p* ≤ 0.01, ***p ≤ 0.001.

### Association of PD-L1 expression with CD8 TIL and clinical outcomes

Univariate analysis of standard prognostic factors in the entire cohort confirmed tumor size, nodal status, ER status, PR status and high tumor grade as significant prognostic factors (Supplementary Table 3). PD-L1 and eCD8 and sCD8 were also not prognostic for relapse free survival (RFS) or overall survival (OS) in the overall cohort (Supplementary Table 3) or in any of the non-triple negative molecular subtypes (data not shown). Within the combined group of Triple Negative breast cancers (TNNB and Basal-like subsets), PD-L1 positive status showed a trend toward better OS and was significantly associated with RFS; in contrast, eCD8 TIL positive status was not associated with RFS or OS (data not shown). In the TNNB subset, PD-L1 positive status and CD8 positive status were also not significant for RFS or OS.

However, in the Basal-like subset, PD-L1 positive status was significantly associated with RFS (Hazard ratio = 0.39, 95% CI = 0.22 - 0.86, *p* = 0.018) and showed a trend toward better OS (Figure 2 and Supplementary Table 3). Consistent with our prior report [24], eCD8 positive status was significant for RFS and OS within the Basal-like subset (Hazard ratio = 0.41, 95% CI = 0.20 - 0.87, *p* = 0.021) (Figure 2). When PD-L1 and eCD8 status were considered together, tumors that were positive for both PD-L1 and eCD8 were associated with RFS and OS, whereas tumors with mixed status or dual negative PD-L1/eCD8 status were not (Hazard ratio = 0.12, 95% CI = 0.10 - 0.71, *p* = 0.010 and Hazard ratio = 0.11, 95% CI = 0.11 - 0.68, *p* = 0.006 respectively) (Figure 2 upper panels and Table 3). Within the Triple Negative group, this association remained significant in the Basal-like subset but not the TNNB subset (Figure 2 middle and lower panels) and was the only prognostic parameter tested that was significant (Table 3).

**Table 3:** Univariate and multivariate analyses of associations between clinical parameters and PD-L1/eCD8 combined status and either relapse free survival or overall survival in the Basal-like subgroup

A/Recurrence-free survival		Univariate		Multivariate	
Parameter	Comparison	HR (95% CI)	*p*-value	HR (95% CI)	*p*-value
Age at diagnosis (yrs)	>35 vs ≤35	0.95 (0.22-4.14)	0.947	0.26 (0.05-1.47)	0.127
Tumor size (cm)	>2cm vs ≤2cm	2.33 (0.79-4.94)	0.151	1.86 (0.50-6.93)	0.354
Nodal status	pos vs neg	1.45 (0.64-3.25)	0.380	1.44 (0.54-3.82)	0.463
Tumor grade	2 vs 1	0.39 (0.10-2.22)	0.349	0.44 (0.05-3.67)	0.450
	3 vs 1	1.86 (0.35-7.69)	0.536	1.18 (0.44-3.21)	0.743
PD-L1 + eCD8 expression	Both high vs both low	0.12 (0.10-0.71)	**0.010**	0.10 (0.01-0.96)	**0.046**
**B/Overall survival**		**Univariate**		**Multivariate**	
**Parameter**	**Comparison**	**HR (95% CI)**	***p*-value**	**HR (95% CI)**	***p*-value**
Age at diagnosis (yrs)	>35 vs ≤35	0.96 (0.22-4.14)	0.954	0.13 (0.02-0.91)	0.040
Tumor size (cm)	>2cm vs ≤2cm	1.74 (0.66-3.96)	0.294	1.20 (0.36-3.98)	0.764
Nodal status	pos vs neg	1.42 (0.65-3.06)	0.393	1.21 (0.47-3.08)	0.693
Tumor grade	2 vs 1	0.41 (0.10-2.44)	0.393	0.40 (0.05-3.29)	0.396
	3 vs 1	2.08 (0.41-7.50)	0.459	1.12 (0.43-2.92)	0.824
PD-L1 + eCD8 expression	Both high vs both low	0.11 (0.11-0.68)	**0.006**	0.07 (0.06-0.75)	**0.028**

To validate these findings, we conducted in-silico analysis of microarray gene expression data using an online survival analysis tool to assess the prognostic effect of PD-L1 in another cohort [26]. Supplementary Figure 1 shows that PD-L1 was prognostic for RFS but not OS within this overall cohort (RFS: *p* < 0.001, OS: *p* = ns). Within the Basal-like subset, PD-L1 was prognostic for both RFS and OS (RFS: *p* = 0.0001; OS: *p* = 0.011).

## DISCUSSION

In this study, we used the SP142 rabbit monoclonal antibody that recognizes an epitope in the C-terminus of human PD-L1 protein and an empirically selected cutpoint that corresponds to the upper quartile of expression levels in the cohort to determine PD-L1 tumor cell expression status and its relation to outcome in breast cancer subsets. PD-L1 was positive overall in 16.5% of a typical consecutive series of breast tumors and was associated with high TIL and with better survival outcome only in the Basal-like subtype.

In assessing the literature published to date regarding tumor cell expression of PD-L1 and outcomes in breast cancer (see Supplementary Table 1), it is important to consider several factors that vary between studies, including antibodies, scoring cut-off definitions, the composition of the cohorts and assessment methods. Different retrospective studies have reported PD-L1 breast tumor cell expression in 21% to 64% of cases but have used a spectrum of different approaches to assess expression [16, 17, 20–23, 27–29]. Amongst those studies that have used an immunohistochemistry approach that can discriminate between tumor cell and host immune cell expression, several different antibodies and cutpoints have also been used [16, 17, 21–23, 27]. It is also important to note that while several of the latter studies reported on analysis of Basal-like tumors, the categorization approach described in some of these studies was incomplete (e.g. subtyping on clinical ER/PR and Her2 status alone [17, 27]) and in these studies the cases described as Basal-like would actually correspond to what we have defined here as the Triple Negative subset. Only one comparable immunohistochemistry study (with a substantial sized cohort of basal-like cases defined by the same 5 biomarker panel but using a different PD-L1 antibody), has recently been published and made similar observations to those in the comparably sized primary and larger validation cohorts used in this study [22]. However, these findings should be qualified by the relatively small Basal-like cohorts in both studies that may in part account for the fact that none of the standard clinical prognostic variables, including nodal status, were statistically significant for outcomes in the basal-like subsets in either study. Also it should be noted that the cohort in this study was selected in order to provide long term outcome data but predates current surgical approaches and adjuvant therapies that have improved outcomes.

Although the majority of Triple Negative breast cancers have Basal-like characteristics (and the majority of tumors expressing ‘basal’ markers are triple-negative), these two terms are not synonymous [30]. Several groups have used genomic analysis to identify different subsets within Triple Negative cancers including some that are delineated by gene expression profiles suggesting weak versus strong intratumoral immune responses and that differ in prognosis [31, 32]. For example Burstein et al focusing specifically on the basal-like subtype delineated four subgroups: (i) luminal androgen receptor (AR; LAR), (ii) mesenchymal (MES), (iii) basal-like immunosuppressed (BLIS), and (iv) basal-like immune-activated (BLIA) subsets, each with distinct characteristics [33]. The BLIS subgroup appears to correspond to cluster C2 [34] and overlaps with the mesenchymal subgroup defined by others [35], while the BLIA subgroup is reflected in the cluster C3 described in other studies [34]. It is interesting that the BLIA subtype displays upregulation of genes associated with B cell, T cell, and natural killer cells. Accordingly, it also exhibits activation of STAT transcription factor–mediated pathways and has the best relative prognosis [32]. It is possible that the BLIA subtype may correspond to the PD-L1 positive/TIL high tumors observed here to exhibit a relatively better prognosis. The BLIA subgroup may also be mirrored by the Immunity 2 metagene signature [36]. Interestingly this metagene signature is highly correlated with PD1 and PD-L1 metagenes and also a good prognosis.

PD-L1 is expressed by many cell types and in the tumor microenvironment PD-L1 is predominantly expressed by CD68 macrophages [15]. But it is well documented that PD-L1 is also expressed by tumor cells and can inhibit the antitumor activity of CD4+ and CD8+ T cells via the inhibitory receptor PD-1 [3, 4]. PD-L1 has therefore generally been assumed to act as an immunosuppressive molecule and indeed has been associated with diminished TIL and poor prognosis in a range of malignancies [7–11]. In contrast, our results and those of others suggests that expression of tumor PD-L1 positively correlates with higher TIL [37, 38], a better response to neoadjuvant chemotherapy [39], and improved overall outcomes in some types of breast cancer [20–23]. While it may seem paradoxical that an immunosuppressive molecule should show these favorable associations, it is now recognized that TIL can induce tumor cell PD-L1 expression by producing cytokines such as interferon gamma (IFN γ) [40]. Indeed, IFN γ-induced PD-L1 expression has been demonstrated in Basal-like subtype breast cancer cell models along with other types of cancer cells [18, 41]. This phenomenon has been referred to as “adaptive resistance”, as it represents one potential means by which tumors can oppose immune infiltration with corresponding anti-tumor cell activity [42, 43]. However, while the effectiveness of this adaptive resistance mechanism may be limited, the induction of PD-L1 remains an indicator of strong antitumor immunity within the tumor (infiltrating lymphocytes producing cytokines in response to tumor-specific or tumor-selective antigens). The positive association between TIL and PD-L1 expression described here is consistent with this model and is further supported by our observation that tumors positive for both PD-L1 and CD8 TIL had better outcomes than those positive for PD-L1 alone within the Basal-like subtype. However, adaptive resistance may not be the only factor in driving PD-L1 expression as it can be up-regulated in breast cancer by other mechanisms such as PTEN loss and ensuing activation of the PI3K pathway [37].

In conclusion, these observations are consistent with the view that PD-L1 expression in tumor cells represents an adaptive immune resistance mechanism and is associated with a better prognosis. Moreover, our results suggest that Basal-like breast cancers may respond best to PD1/PD-L1 blockade.

## MATERIALS and METHODS

### Case cohort

A cohort of 443 breast cancer cases was studied representing primary tumors collected by the Manitoba Breast Tumor Bank [44] at time of diagnosis and initial surgical intervention. Age at diagnosis, tumor grade, size, nodal status, and outcomes in terms of relapses and deaths were recorded. All tumors were histologically classified and graded by one pathologist (PHW). The time of diagnosis and accrual by the bank (1988-1995) predated current biomarker assays. Therefore immunohistochemistry (IHC) was previously performed by the Bank using an auto-immunostainer (Discovery Staining Module, Ventana Medical Systems, AZ, USA) on TMA sections from the cohort for ER, PR, Ki67, CK5/6, EGFR and Her2 biomarkers. ER, PR, and Her2 were scored and positive status assigned according to ACP guidelines [45, 46]. Ki67, CK5/6 and EGFR were also scored and positive status assigned as >14% (Ki67) or any positive tumor cell staining (CK5/6 and EGFR). On the basis of the IHC determined expression of these five biomarkers the cohort was classified by the Bank into five intrinsic molecular subtypes: Luminal A (ER+/Ki67-/Her-), Luminal B (ER+/Ki67+/Her-), Her2 (Her2+), Triple Negative Non-Basal (TNNB, ER-/PR-/Her-/CK5/6-/EGFR-), and Basal-like (ER-/PR-/Her- and either CK5/6+ and/or EGFR+) [47, 48]. The Tumor Bank operates with approval of the University of Manitoba Biomedical Research Ethics Board and this research study was conducted under approval from the BC Cancer Agency Research Ethics Board. A report concerning the source of the biospecimens and data used according to the BRISQ guidelines [49] is provided in Supplementary Table 2.

### Tissue microarray (TMA) construction

Primary tumors were represented in tissue microarrays (TMAs) compiled by the Tumor Bank. To construct a TMA, all cases were initially selected from the database and then sections were re-reviewed to confirm and select areas for coring of corresponding blocks. Duplicate tissue cores (0.6 mm diameter) were taken from central cellular areas of each tumor with a tissue arrayer instrument (Beecher Instruments, Silver Spring, MD, USA). The original cohort of 636 cases was arrayed across 7 blocks. Prior utilization of these blocks and exhaustion of individual cores meant that the final interpretable cohort for this study was 443 cases.

### Immunohistochemistry and TMA scoring

PD-L1 and CD8, CD4, CD68 staining was performed on deparaffinized sections from TMAs using a Biocare Medical Intellipath FLX autostainer using reagents from Biocare (Concord, CA) unless otherwise noted. Slides were deparaffinized manually through xylene and graded alcohols then antigen retrieval performed in Biocare's decloaking chamber using Diva decloaking solution for 125°C for 30 seconds. Slides were loaded into the Intellipath FLX, subjected to non-specific blocking with Peroxidased-1 and background sniper then incubated with either PD-L1 (clone SP142, Pleasanton, CA, 1/1000), CD8 (clone C8/144B, Cell Marque, Rocklin, CA, 1/250), CD4 (clone EPR6855, Abcam, 1/250) or CD68 (clone SP251, Spring Biosciences, Pleasanton, CA, 1/150) in Da Vinci Green diluents for 30 minutes at room temperature. The slides were then incubated with either Rabbit-(PD-L1, CD68, CD4)-HRP or Mach2 Mouse-(CD8) polymer for 30 minutes at room temperature and then detected with IP DAB for 5 minutes followed by counterstaining with a 1:10 dilution of CAT hematoxylin, air drying and coverslipping with Ecomount.

IHC scoring was performed by an experienced breast pathologist (PHW) in a blinded fashion with respect to each case. TMA sections were initially assessed at low magnification to select the core with the highest density of positive cells. PD-L1 expression by tumor cells was then assessed in the area of the 0.6 mm core by the H score method whereby the expression is quantitated as the product of staining intensity (ranked from 0 to 3) and proportion of positive staining tumor cells (0 to 100%) to give an expression score range from 0 to 300. PD-L1 expression in TIL was not scored. For statistical analyses, tumor cell expression was categorized into low or high expression levels based on scores below or above the upper 75^th^ quartile. TIL markers were assessed as described previously [50] by direct counting of positive cells (numbers were based on exact counts up to 20 cells or estimated when cell numbers were in excess of this number (IHC score, range 0–100). The area of the entire core occupied by tumor epithelium versus stroma was then assessed followed by estimation of the proportion of positive TIL that were intra-epithelial or intra-stromal; intra-epithelial localization was defined as lymphocytes within tumor cell nests and/or in direct contact with tumor cells. Intra-epithelial and intra-stromal TIL density per core was then calculated for each TIL subset (and designated, for example, as eCD8 and sCD8), and duplicate values were averaged for each case. For statistical analysis CD8 TIL levels in tumors were categorized into low or high TIL status based on the upper quartile (75^th^ percentile) of epithelial and stromal TIL- density scores (eCD8 and sCD8).

### Statistical analysis

Associations between PD-L1 expression and clinical-pathological features were evaluated using chi-square test and, where necessary, Fisher t test. Comparisons of intra-epithelial and intra-stromal TIL densities stratified by PD-L1 high/low expression were performed using Mann-Whitney test. Assessment of the correlation between PD-L1 and other immune markers was performed using a nonparametric Spearman correlation. Survival was calculated using the Kaplan-Meier method, and curves were compared with the log-rank test. Multivariate survival analyses were done using Cox regression analysis. All statistical tests were two-sided with significance established at *p*-values less than 0.05. Statistical analyses were performed using Graphpad Prism 6.0 (GraphPad, La Jolla, CA, USA) and SPSS statistics 17 (SPSS, Chicago, IL, USA).

## SUPPLEMENTARY MATERIALS FIGURES AND TABLES






