# Supplementary material for: PD-L1 and intratumoral immune response in breast cancer
Source: Oncotarget. 2017 May 30;8(31):51641–51. doi: 10.18632/oncotarget.18305 (PMC5584276; doi:10.18632/oncotarget.18305)
Supplement: Supplementary file 1 [file oncotarget-08-51641-s001.pdf]

# PD-L1 and intratumoral immune response in breast cancer

## SUPPLEMENTARY MATERIALS

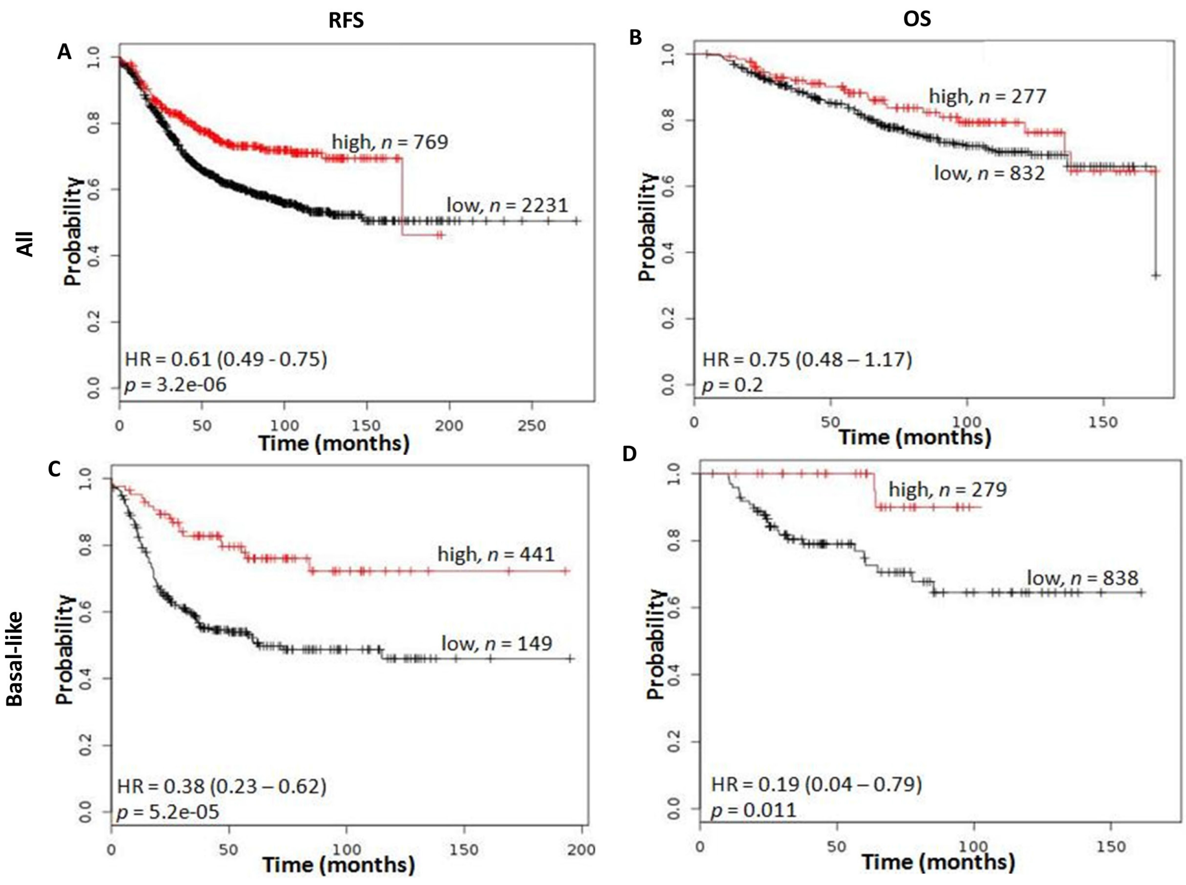


**Supplementary Figure 1: The relation between PD-L1 and RFS (left column) and OS (right column) was analyzed using the kmplotter tool.** RFS and OS were assessed in all cases (panels **A** & **B**) and Basal-like subgroup (panels **C** & **D**) using the Jetprobe set for PD-L1 (Affymetrix ID: 227458) and a cutpoint at the 75th expression quartile. Kaplan Meier plots are shown with hazard ratios (HR) and logrank test *p* values.

**Supplementary Table 1: Reported studies of PD-L1 expression in different breast cancer studies**

See Supplementary File 1

**Supplementary Table 2: A report concerning the source of the biospecimen and data using the BRISQ guidelines [49]**

See Supplementary File 2

**Supplemental Table 3: Univariate and multivariate analyses of clinical parameters and PD-L1 or eCD8 status and their relation with recurrence free survival and overall survival in all cases**

| A/ Recurrence-free survival | | Univariate | | Multivariate | |
|---|---|---|---|---|---|
| **Parameter** | **Comparison** | **HR (95% CI)** | ***p*-value** | **HR (95% CI)** | ***p*-value** |
| Age at diagnosis (yrs) | > 35 vs ≤ 35 | 0.48 (0.13-0.94) | **0.038** | 0.73 (0.27-1.97) | 0.530 |
| Tumour size (cm) | > 2cm vs ≤ 2cm | 2.17 (1.42-2.64) | **< 0.0001** | 2.14 (1.37-3.34) | **0.001** |
| Nodal status | pos vs neg | 2.03 (1.52-2.70) | **< 0.0001** | 1.91 (1.35-2.71) | **0.000** |
| Tumor grade | 2 vs 1 | 1.49 (0.97-2.10) | 0.070 | 0.99 (0.53-1.84) | 0.965 |
| | 3 vs 1 | 1.84 (1.14-2.84) | **0.012** | 1.39 (0.87-2.24) | 0.170 |
| ER status | pos vs neg | 0.44 (0.30-0.54) | **< 0.0001** | 0.53 (0.36-0.79) | **0.002** |
| PR status | pos vs neg | 0.46 (0.34-0.60) | **< 0.0001** | 0.52 (0.36-0.76) | **0.001** |
| Molecular subtypes | | | | | |
| Luminal A | PD-L1 high vs low | 0.99 (0.47-2.06) | 0.972 | | |
| Luminal B | | 1.26 (0.45-3.72) | 0.638 | | |
| Her2 | | 1.31 (0.43-4.31) | 0.602 | | |
| TNNB[a] | | 1.07 (0.75-1.54) | 0.689 | | |
| Basal-like | | 0.39 (0.22-0.86) | **0.018** | | |
| eCD8 expression | high vs low | 1.07 (0.75-1.54) | 0.745 | 1.00 (0.99-1.00) | 0.245 |
| PD-L1 expression | high vs low | 0.86 (0.60-1.27) | 0.470 | 0.96 (0.58-1.58) | 0.866 |

| B/ Overall survival | | Univariate | | Multivariate | |
|---|---|---|---|---|---|
| **Parameter** | **Comparison** | **HR (95% CI)** | ***p*-value** | **HR (95% CI)** | ***p*-value** |
| Age at diagnosis (yrs) | > 35 vs ≤ 35 | 0.47 (0.11-0.97) | **0.045** | 1.15 (0.39-3.36) | 0.804 |
| Tumour size (cm) | > 2cm vs ≤ 2cm | 1.76 (1.17-2.29) | **0.0036** | 1.77 (1.13-2.79) | **0.013** |
| Nodal status | pos vs neg | 1.75 (1.29-2.37) | **0.0004** | 1.56 (1.07-2.26) | **0.019** |
| Tumor grade | 2 vs 1 | 1.64 (1.02-2.33) | 0.043 | 0.81 (0.42-1.57) | 0.531 |
| | 3 vs 1 | 2.64 (1.55-3.89) | **0.0001** | 1.30 (0.80-2.10) | 0.287 |
| ER status | pos vs neg | 0.32 (0.20-0.37) | **< 0.0001** | 0.36 (0.24-0.55) | **0.000** |
| PR status | pos vs neg | 0.37 (0.27-0.48) | **< 0.0001** | 0.48 (0.32-0.72) | **0.000** |
| Molecular subtypes | | | | | |
| Luminal A | PD-L1 high vs low | 0.93 (0.41-2.15) | 0.875 | | |
| Luminal B | | 1.11 (0.36-3.44) | 0.851 | | |
| Her2 | | 1.90 (0.71-8.07) | 0.164 | | |
| TNNB[a] | | 1.16 (0.80-1.71) | 0.415 | | |
| Basal-like | | 0.51 (0.28-1.04) | 0.067 | | |
| eCD8 expression | high vs low | 1.16 (0.80-1.71) | 0.415 | 1.00 (0.99-1.00) | 0.221 |
| PD-L1 expression | high vs low | 1.02 (0.68-1.52) | 0.936 | 1.17 (0.71-1.94) | 0.540 |

[a]TNNB = Triple Negative Non-basal.
