# Supplementary material for: PD-L1 and intratumoral immune response in breast cancer
Source: Oncotarget. 2017 May 30;8(31):51641–51. doi: 10.18632/oncotarget.18305 (PMC5584276; doi:10.18632/oncotarget.18305)
Supplement: Supplementary file 3 [file oncotarget-08-51641-s003.docx]

**Supplementary Table 2**. **A report concerning the source of the biospecimen and data using the BRISQ guidelines [49]**

| BRISQ Report ID : 40b |
| --- |
| **Profile**  Study Name : PD-L1  Biobank Name : Manitoba Breast Tumor Bank  Datetime Created : 20-Oct-2016 |
| **Pre-Acquisition**  Biospecimen Type : Tissue (not otherwise specified)  Anatomical Site : breast  Disease Status of Participants : primary cancer specimens from breast cancer patients  Clinical Characteristics Of Participants : invasive breast cancer  Vital State Of Participants : alive  Disease State : outcomes data provided by Manitoba Breast Tumor Bank  Cause of Death : n/a  Agonal State : n/a  Diagnosis : invasive breast cancer  Clinical : primary breast cancer  Pathology : invasive carcinoma, NOS  Time Between Diagnosis And Sampling : typical pathology service times, 0.5 - 2 hrs  Exposures : none  Reproductive Status : unknown  Patient Demographic Information : female  Accrual Scheme : tumor bank generalized collection scheme....all available breast cancer specimens  Nature Biobanking Institutions : Manitoba Breast Tumor Bank |
| **Acquisition**  Collection Mechanism : surgical resection specimens  Time From Cessation Blood Flow In Vivo To Biosepecimen Excision : unknown  Time From Biospecimen Excision Acquisition To Stabilization : typical pathology service times, 0.5 - 2 hrs  Temperature Between Biospecimen Excision Acquisition and Stabilzation : RT  Collection Container : unknown |
| **Stabilization and Preservation**  Mechanism of Stabilization : frozen specimens, prior to conversion to FFPE blocks  Type of Long Term Preservation : formalin fixed paraffin embedded  Constitution of Preservative : 10% neutral buffered formalin  Time in Fixative Preservation Solution : unknown  Temperature During Time in Preservation Solution : unknown  Aliquot Volume : n/a  Specimen Size : typical clinical blocks |
| **Storage and Handling**  Storage Temperature : room temp  Storage Duration : up to 20 yrs  Storage Details : unknown  Type of Storage Container : unknown  Type of Slide : charged glass for IHC  Shipping Parameters : unknown  Shipping Duration : unknown  Type of Transport Container : unknown  Shipping Temperature : room temp  Freeze Thaw Parameters :  Number of Freeze Thaw Cycles : n/a  Duration of Thaw Events : all FFPE blocks created from previously frozen specimens by immersion of frozen aliquot in formalin  Time from Last Thaw to Processing : unknown |
| **Quality Assurance**  Composition Assessment and Selection : detailed histology composition analysis of section  Gross and Microscopic Review : comprehensive; conducted by a single pathologist  Proximity to Primary Pathology of Interest : tumor central portion  Method of Enrichment for Relevant Component : TMA coring, targeting central region of tumor in block  Details of Enrichment for Relevant Component : n/a  Quality Assurance Measures : n/a  Embedding reagent/medium : unknown |
